# Addressing Antivaccine Sentiment on Public Social Media Forums Through Web-Based Conversations Based on Motivational Interviewing Techniques: Observational Study

**DOI:** 10.2196/50138

**Published:** 2023-11-14

**Authors:** David Scales, Lindsay Hurth, Wenna Xi, Sara Gorman, Malavika Radhakrishnan, Savannah Windham, Azubuike Akunne, Julia Florman, Lindsey Leininger, Jack Gorman

**Affiliations:** 1 Weill Cornell Medicine New York City, NY United States; 2 Critica Bronx, NY United States; 3 Tuck School of Business Dartmouth College Hannover, NH United States

**Keywords:** anti-vaccine, digital environment, engagement, health misinformation, infodemic, infodemiology, information environment, medical misinformation, misinformation, observational study, social media engagement metrics, social media

## Abstract

**Background:**

Health misinformation shared on social media can have negative health consequences; yet, there is a dearth of field research testing interventions to address health misinformation in real time, digitally, and in situ on social media.

**Objective:**

We describe a field study of a pilot program of “infodemiologists” trained with evidence-informed intervention techniques heavily influenced by principles of motivational interviewing. Here we provide a detailed description of the nature of infodemiologists’ interventions on posts sharing misinformation about COVID-19 vaccines, present an initial evaluation framework for such field research, and use available engagement metrics to quantify the impact of these in-group messengers on the web-based threads on which they are intervening.

**Methods:**

We monitored Facebook (Meta Platforms, Inc) profiles of news organizations marketing to 3 geographic regions (Newark, New Jersey; Chicago, Illinois; and central Texas). Between December 2020 and April 2021, infodemiologists intervened in 145 Facebook news posts that generated comments containing either false or misleading information about vaccines or overt antivaccine sentiment. Engagement (emojis plus replies) data were collected on Facebook news posts, the initial comment containing misinformation (level 1 comment), and the infodemiologist’s reply (level 2 reply comment). A comparison-group evaluation design was used, with numbers of replies, emoji reactions, and engagements for level 1 comments compared with the median metrics of matched comments using the Wilcoxon signed rank test. Level 2 reply comments (intervention) were also benchmarked against the corresponding metric of matched reply comments (control) using the Wilcoxon signed rank test (paired at the level 1 comment level). Infodemiologists’ level 2 reply comments (intervention) and matched reply comments (control) were further compared using 3 Poisson regression models.

**Results:**

In total, 145 interventions were conducted on 132 Facebook news posts. The level 1 comments received a median of 3 replies, 3 reactions, and 7 engagements. The matched comments received a median of 1.5 (median of IQRs 3.75) engagements. Infodemiologists made 322 level 2 reply comments, precipitating 189 emoji reactions and a median of 0.5 (median of IQRs IQR 0) engagements. The matched reply comments received a median of 1 (median of IQRs 2.5) engagement. Compared to matched comments, level 1 comments received more replies, emoji reactions, and engagements. Compared to matched reply comments, level 2 reply comments received fewer and narrower ranges of replies, reactions, and engagements, except for the median comparison for replies.

**Conclusions:**

Overall, empathy-first communication strategies based on motivational interviewing garnered less engagement relative to matched controls. One possible explanation is that our interventions quieted contentious, misinformation-laden threads about vaccines on social media. This work reinforces research on accuracy nudges and cyberbullying interventions that also reduce engagement. More research leveraging field studies of real-time interventions is needed, yet data transparency by technology platforms will be essential to facilitate such experiments.

## Introduction

Extensive research has shown that health misinformation has real, negative consequences. It can influence people to hold misperceptions and adopt unhealthy behaviors [1-8]. This led the US Surgeon General to issue a special advisory on the topic [9], in which health misinformation was defined as “information that is false, inaccurate, or misleading according to the best available evidence at the time” [10,11]. We also know that although supplying facts is often necessary to counteract misinformation, it is usually not sufficient to change opinions or behavior [12,13]. Belief in misinformation can be deeply ingrained, reinforced by psychological and social pressures, and difficult to dislodge [14]. This is especially the case with information on the internet and social media presented in misleading contexts and subjected to repeated sharing, reposting, and commenting. Some such information, whether true or false, can be spread with the intent to deliberately create misperceptions or sway public opinion [15].

From the onset of the COVID-19 pandemic, the World Health Organization (WHO) described the situation of a parallel “infodemic” [16], defined as “excess information, including false or misleading information, in digital and physical environments during an acute public health event” [17]. This infodemic focus has renewed interest in “infodemiology,” the epidemiological study of these digitally enabled flows of information [18], and the need for professionals equipped to assess and respond to misinformation of public health importance as a core function of public health [19,20]. While an epidemic metaphor has its limits and externalities [21], it offers a framework with which to marshal resources toward understanding and mitigating the problem. Put another way, the lens of an infodemic suggests the need to develop field epidemiologists to deploy in public health and infodemic emergencies for rapid support of public health communications and interventions [22].

While public health institutions such as the Centers for Disease Control and Prevention (CDC) or the WHO issue messages on social media, these public health broadcasts are often at the periphery of web-based discussions about vaccines, Ebola, and the Zika virus [23-25]. Similarly, during the COVID-19 pandemic, information with public health relevance was decreasingly reliant on top-down recommendations from doctors and public health institutions (eg, the CDC) and more reliant on socially contextualized, decentralized, interpersonal, horizontal, and networked communication like that found on social media [26]. In contrast, antivaccine advocates are often leveraging the affordances of digital platforms to communicate in a coordinated, networked fashion [25,27]. We therefore hypothesized that best practice public health recommendations would not speak for themselves but would require trusted, community-linked advocates to communicate and interpret them within the value frames of those networks. We therefore developed a protocol based on principles of motivational interviewing and other evidence-based approaches, including inoculation, use of narratives, and promoting critical thinking, to address misinformation in web-based contexts and used it to intervene on Facebook (Meta Platforms, Inc) when misinformation about COVID-19 vaccines appeared [22].

Our approach is based on a menu of tactics derived from 3 main strategies: assessing how receptive the person posting health misinformation may be to an intervention; increasing high-quality, science-based messages across the web-based communication network; and reducing misinformation across that network. A critical principle underlying the protocol is derived from motivational interviewing (MI) techniques [28], which have shown efficacy in addressing vaccine hesitancy [29]. Using MI principles in this setting meant that the interventionist attempted to establish common ground with the person who posted misinformation and expressed empathy and an interest in understanding their point of view before responding directly to misinformed comments. Open-ended questioning and reflective listening in the spirit of MI are used throughout. Work to fully adapt MI to this setting, which we term community-oriented motivational interviewing, is ongoing [30].

As the person posting misinformation on social media is often committed to the misinformed point of view and unlikely to be immediately persuaded to consider an alternative perspective, the infodemiologists also consider the perspective of “bystanders” to the conversation, those observing but not necessarily engaging or commenting [31]. Such “bystanders” are hypothesized to be part of the “moveable middle” [32] and more persuadable about issues such as COVID-19 vaccines than the initial commentator. As misinformation can be perceived as true through repetition, infodemiologists seek to disrupt that “illusory truth effect” [33] while also role modeling how community members can make decisions commensurate with their values despite scientific uncertainty.

The purpose of this study is to provide a detailed description of infodemiologists’ interventions. We also present an outline of an initial evaluation framework for such work, highlighting major gaps in the lack of accessibility of social media data that hinder researchers’ ability to tie their work to more concrete outcomes, like behaviors. Finally, we quantify the impact these in-group messengers have on the web-based threads on which they are intervening.

## Methods

### Overview

Infodemiologists were drawn from the communities in which they intervene to help ensure trust through shared identity and values [34-37]. Details on the recruitment, credentials, training, and supervision of the 4 infodemiologists involved in this report have been published previously, along with descriptions of the intervention process [22,30]. Briefly, infodemiologists underwent a skills-based training consisting of practice interventions and weekly supervision sessions with one of the authors (DS or JMG) for feedback, totaling approximately 20-30 hours of training, practice, and supervision. They were first assigned independent reading to provide guidance on the evidence behind different communication techniques and then conducted a series of web-based training interventions with supervision, reflection, and feedback with DS and JMG [13,38,39]. The instructions on how to conduct interventions were broad, emphasizing that they needed to be tailored to the context. After the initial training, supervision continued at weekly group reflection sessions with all infodemiologists throughout the course of the study. All infodemiologist interventions were included in data collection and analysis, and none were excluded. A total of 145 pilot interventions were conducted between December 2020 and April 2021.

**Figure 1:**
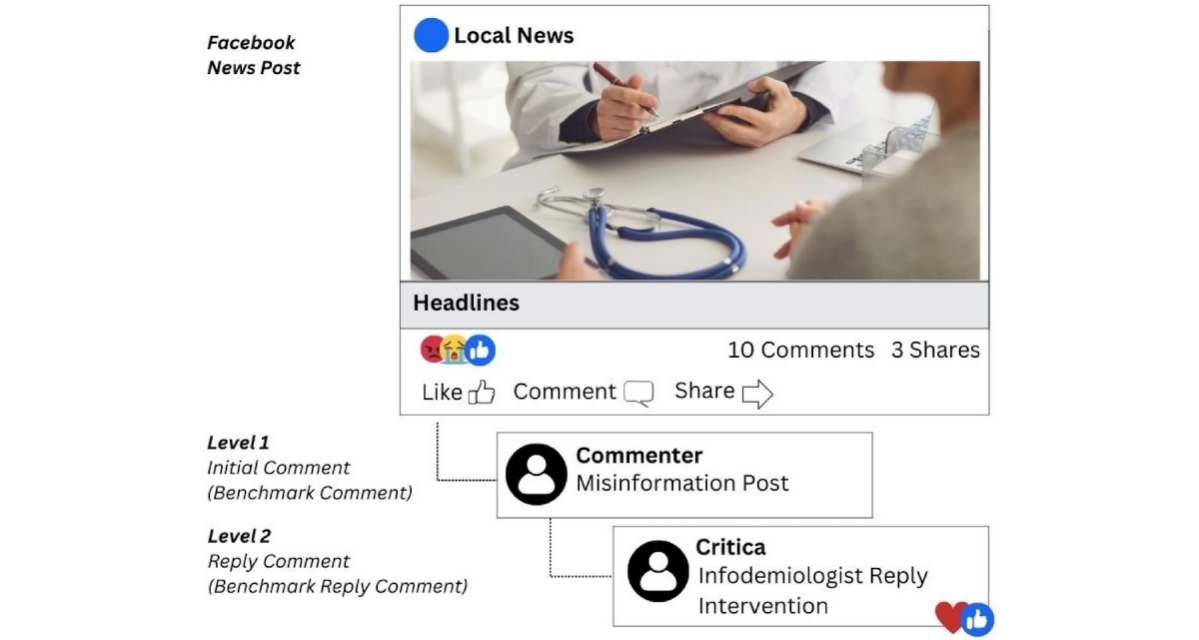
Visual description of comment and reply levels.

**Figure 2:**
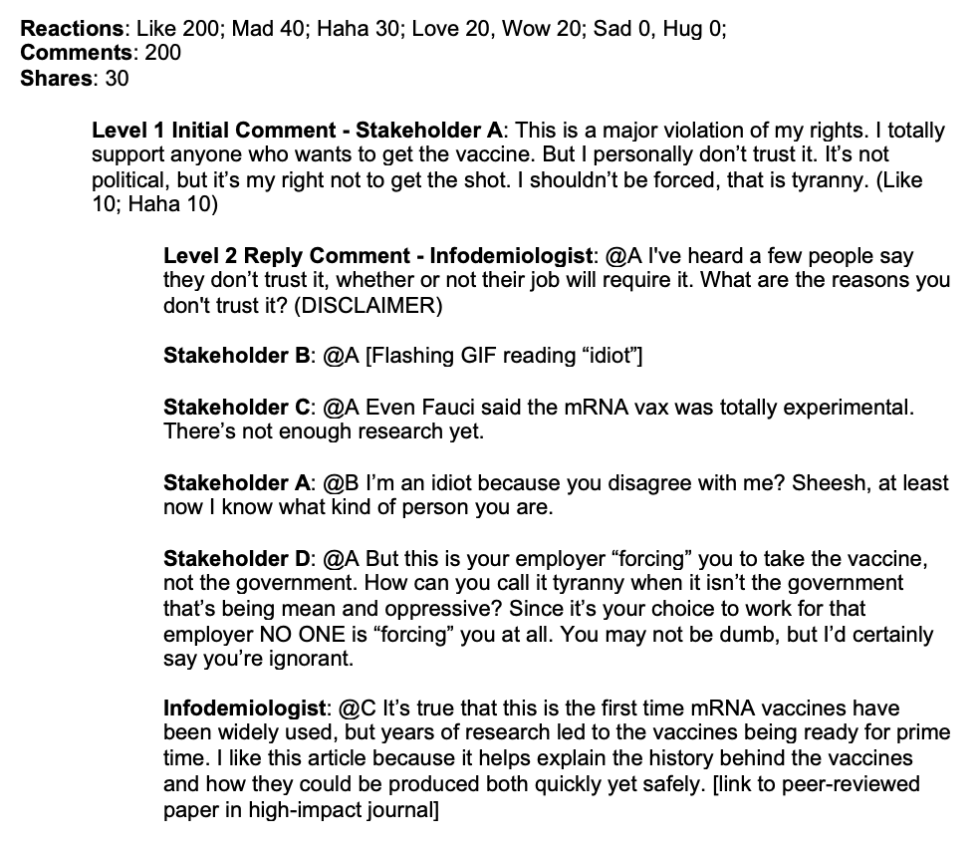
Example transcript of an infodemiologist intervention on a local news article from Texas describing how employers could require COVID-19 vaccines. To prevent retroactive identification, engagement and response numbers are rounded and the transcript paraphrased.

Full details on our misinformation monitoring and identification process are available from Gorman and Scales [22] and Scales et al [30]. In short, we monitored web-based Facebook profiles of news organizations marketing to 3 geographic regions: Newark, New Jersey; Chicago, Illinois; and central Texas. Regions were chosen for demographic, geographic, and urban or rural diversity. Infodemiologists were trained to select local media postings on Facebook that had generated comments containing either misinformation about vaccines or antivaccine sentiment within several hours of their posting. We defined misinformation about COVID-19 vaccines practically as any post that contained factually incorrect material or overtly negative sentiment about the vaccines, regardless of the motive of the person posting. This was a subjective assessment based on the infodemiologist’s perception of what could be considered negative from the perspective of their community. For more details on how threads were chosen for interventions, see [30]. Infodemiologists recorded deidentified transcripts of the conversations (including ancillary comments from bystanders) as well as native engagement metrics (likes, shares, etc). Information on matched comments and replies for benchmarking was collected later, but sensitivity analysis did not find significant changes in conversation metrics over time. Infodemiologists were supported in their work through a process of written reflection after each intervention, direct written feedback on their interventions, and weekly group supervision sessions. Moreover, to protect them from harassment, infodemiologists were instructed to exit conversations that became emotionally heated or where they felt unsafe. To minimize web-based harassment, infodemiologists used the Critica’s Facebook account and only identified themselves by their first names. The structure of the comments can be found in Figure 1, and an example of an intervention can be seen in Figure 2, paraphrased to protect the privacy of participants [40].

The infodemiologist intervened on a local news article from Texas describing how employers could require COVID-19 vaccines. To ensure the original post cannot be retroactively identified, the engagement and response numbers are rounded, and the transcript has been paraphrased.

In any given infodemiology session, infodemiologists deployed various evidence-informed communication techniques depending on the context (Table 1). Since there is little evidence to guide what communication technique should be used at any given time, we developed a 2-pronged approach to guide how and when to apply different intervention techniques. We engaged in discursive reflection among our team members, sometimes in real time through email or Slack (Slack Technologies) channels and at our weekly reflection meetings, to assess what techniques appeared to curtail conversations and engagement or promote reflection or resistance (ie, “change talk” or “sustain talk” per motivational interviewing language). Additionally, we paid particular attention to whether interventions elicited backfire effects, or psychological reactance, defined as escalating negative emotions through the course of an interaction with the infodemiologist [41]. Immediately after every initial infodemiologist intervention, they posted a disclaimer identifying themselves as researchers and a web link to further information about the research study, including options to request data be removed from our database. Of note, no requests for data removal were received. Table 1 provides a glimpse into the range of communication techniques that infodemiologists may use and the evidence behind them. It is not a comprehensive compilation of such techniques or the supporting evidence. A full review of this literature is beyond the scope of this study.

**Table 1:** Infodemiologist menu of techniques, protocols, and corresponding evidence.

Principle or approach and goal		Explanation or example	Reference
**Receptivity to finding misinformation credible**			
	Infodemiologists should be from and within the communities and networks in which they will be intervening	Example cues (language, register, and slang) as markers of in-group identity	[35,42,43]
	Assess readiness for change	Precontemplation, contemplation, preparation, action, and maintenance	[44,45]
	Apply relevant principles of motivational interviewing	Open-ended questions, affirmations, reflective listening, summarizing, and promoting self-efficacy	[38,46,47]
	Focus on the “fencers” or the “moveable middle”	People with heavily committed beliefs are unlikely to change their views quickly.	[31,32,48]
	Promote critical thinking	“Please tell me more about that? Maybe give an example?”	[49]
	Inoculation	“Misinformation will use various methods to make you doubt vaccines, like saying, “vaccines will make you infertile forever!”	[35,50-53]
**Increase high-quality, science-based messages across the network**			
	Targeting highly visible Facebook news sources with little comment moderation	Facebook is often slow to implement its own misinformation takedown policies.	[54-56]
	Reframing negative comments according to in-group cultural values regarding uncertainty, freedom to choose, etc	“I understand masks feel to you like they restrict freedom, but I’m proud to wear a mask with traditional designs that also helps protect our elders.”	[57-59]
	Use personal narratives or anecdotes	“I was so relieved when I got vaccinated. I stopped worrying that I’d die if I got COVID”	[60]
	Respond as quickly as possible after comments are posted	Ensures misinformation does not become entrenched or misinterpreted.	[61,62]
	Detailed rebuttals, if needed	Rebuttals without explanations are less effective.	[49,63,64]
	Link to sources likely to be trusted by commenters	Example: not citing the CDC^a^ or the FDA^b^ as sources if stakeholders are antigovernment	[34,61,65]
	Repetition	People attribute more accuracy to repeated information.	[66]
**Reduce misinformation across the network**			
	Reframing uncertainty as congruent with values	Unknown risks psychologically loom large; reframing them around known benefits provides balance.	[57,58]
	Offering alternate, more plausible explanations	Plausible explanations accompanying warnings or rebuttals increase effectiveness.	[67]
	Appeal to expert consensus	“97% of climate scientists agree that human-caused climate change is happening.”	[35,68-70]
	Appeal to accuracy	Asking, “how accurate is that headline?”	[71,72]
	Recontextualizing information taken out of context	“While true, these data are unverified user reports, not official statistics”	[73]

To build an evaluation strategy, we used the reach, effectiveness, adoption, implementation, and maintenance (RE-AIM) framework from implementation science [74], which provided a strategic guide for ideal evaluation of infodemiology interventions. Originally designed to incentivize scientists to be transparent and reflect on internal and external validity across the continuum of translational research from pilot to effectiveness studies, the RE-AIM framework was chosen here for 2 main reasons. First, it is familiar, being one of the most widely used and cited implementation science frameworks. Second, it has been successfully adapted to multiple and diverse contexts, suggesting it could also be applied to the web-based setting in which this research was done [75].

While implementation science is often used for interventions whose efficacy and effectiveness have already been established and which require further intervention to ensure their uptake into a specific context of interest, we believe it offers useful frameworks for work in digital spaces to counter misinformation, even though the evidence base is still emerging. In that context, we note that the communication techniques listed in Table 1 have demonstrated varying degrees of efficacy, and not all have demonstrated effectiveness in real-world, web-based settings. However, due to the constantly changing nature of web-based platforms, we recognized that effectiveness data either would not be forthcoming quickly or would essentially be outdated by publication due to platform changes (algorithmic, graphical user interface, or other). We therefore took the approach outlined by Lane-Fall et al [76] that hybrid implementation-effectiveness studies may be most appropriate when the urgency of the situation requires it, coupled with strong indirect evidence of potential effectiveness in the context of interest. In this study, we focus on the effectiveness results. Refer to Gorman and Scales [22] for an implementation discussion.

We collected data on the Facebook news post, the initial comment containing misinformation, which we will refer to as a “level 1 comment,” and the infodemiologist reply comment, which is the start of the intervention, referred to as a “level 2 reply comment” (Figures 1 and 2 contain visual descriptions and an example of different comments and replies described here). Facebook organizes comments into threads, with level 2 comments branching off level 1 comments. Engagement was defined as the total number of comments and emoji reactions (like, love, hug, mad, haha, wow, and sad). In the context of this topic, emoji reactions “like,” “love,” and “hug” are interpreted as positive reactions, and “mad” and “haha” are negative reactions, with the latter interpreted as sarcasm. “Wow” and “sad” are considered neutral reactions.

We implemented an innovative comparison-group evaluation design, building upon existing designs that measure engagement without a comparative benchmark [77-79]. Specifically, we collected data on comparison comments and replies adjacent to the intervention threads. Comments gathered for benchmarking were the five level 1 comments immediately above and below the level 1 comment to which the infodemiologist replied at the time of subsequent data collection, which, due to Facebook algorithms that are not transparent, may have changed from the time of the intervention. In rare circumstances where the level 1 comment subject to an infodemiologist’s intervention could not be found (eg, absorbed into “Relevant” by Facebook), data were collected on 10 comments from the middle of the comments thread. Reply comments gathered for benchmarking were collected in the same way: the five level 2 comments above and below the infodemiologist’s level 2 reply comment.

Collecting the comments data for benchmarking allowed us to consider the question, “How does engagement on the level 1 comment the infodemiologist chose to respond to compare to the engagement on the typical level 1 comment on the same Facebook news post?” Benchmarking with reply comments data allowed us to compare the engagement the infodemiologist interventions (ie, level 2 reply comments) received to the engagement on the typical level 2 reply comment on the same Facebook news post. For each level 1 comment, we summarized the infodemiologists’ level 2 reply comments into 6 metrics: the median and IQR of the respective numbers of replies, emoji reactions, and engagements infodemiologists’ reply comments received. For comparative purposes, we also summarized the numbers of comments and reply comments and the corresponding 6 metrics for the comments used for benchmarking. The numbers of replies, emoji reactions, and engagements level 1 comments received were compared with the median metrics of matched comments using the Wilcoxon signed rank test. Each metric of infodemiologists’ level 2 reply comments (intervention) was benchmarked against the corresponding metric of matched reply comments (control) using the Wilcoxon signed rank test (paired at the level 1 comment level). The number of replies, emoji reactions, and engagements between infodemiologists’ level 2 reply comments (intervention) and matched reply comments (control) were further compared using 3 Poisson regression models: treating intervention as a fixed effect with the Huber-White robust SE estimates, as a random effect (nested within location), and as a random effect (nested within location) with the number of page followers/1,000,000 as an offset. The fixed effect design is particularly strong as an internal validity test, as it controls for any confounding, both observed and unobserved, across level 1 posts. For example, level 1 posts differed by geography, timing, and news organization. Limiting comparisons to level 2 intervention and treatment posts nested within the same level 1 post sweeps away any of these concerns. The tradeoff to fixed effects estimation is a relative loss of statistical precision and an inability to characterize level 1 influences; as such, we estimated random effects models as a robustness exercise. The significance level was set at =.05, and no correction was made for multiple testing as this was an exploratory and hypothesis-generating analysis.

### Ethical Considerations

This study was deemed exempt from institutional review board (IRB) review by the Salus IRB (#2014) and approved by the Weill Cornell Medicine IRB (20-10022858). To comply with Facebook Terms of Service, all data were manually collected by infodemiologists and manually reviewed for accuracy during a follow-up assessment approximately 10 months later.

## Results

A total of 145 interventions were conducted on 132 Facebook news posts, of which 55 interventions (38%) focused on Illinois news sources, 8 (6%) in New Jersey, 45 (31%) in Texas, and 37 (26%) in other states or were nationally oriented. Two-thirds (93/145, 64.14%) of infodemiologist interventions precipitated some form of engagement (either an emoji reaction or a reply comment) from commenters or activated bystanders. In keeping with related literature, comment engagements were right-skewed. Accordingly, we calculated medians and IQRs. The Facebook page for the news organizations on which infodemiologist interventions (level 2 reply comments) were posted had a median of 915,860 (Q1-Q3 range 634,473-2,689,864) page followers. The Facebook news posts received a median of 19 (Q1-Q3 range 7-86) shares, 119 (Q1-Q3 range 37-352) comments, 190 (Q1-Q3 range 73-510) emoji reactions, and 354 (Q1-Q3 range 119-918) engagements, which are the sum of the numbers of comments and emoji reactions received (Table 2).

**Table 2:** Metrics for Facebook news page and Facebook news post.

	Page followers, median (Q1-Q3)	Shares, median (Q1-Q3)	Comments, median (Q1-Q3)	Reactions, median (Q1-Q3)	Engagements^a^, median (Q1-Q3)
Facebook Page	915,860 (634,473- 2,689,864)	—^b^	—	—	—
Facebook news post	—	19 (7-86)	119 (37-352)	190 (73-510)	354 (119-918)

^a^Engagements = comments + emoji reactions for Facebook news post.

^b^Not available.

The level 1 comments received a median of 3 replies, 3 reactions, and 7 engagements. The matched comments received a median of 0 replies with a median IQR of 0.75, 1 emoji reaction with a median IQR of 2, and therefore 1.5 engagements with a median IQR of 3.75. Compared to the matched comments, the level 1 comments received more replies, emoji reactions, and engagements (Table 3).

**Table 3:** Metrics for level 1 comment and matched comment.

		Level 1 comment, median (Q1-Q3)	Matched comment, median (Q1-Q3)	*P* value^a^	
**Replies**					
	Median	3 (2-7)	0 (0-0.5)	<.001	
	IQR	—^b^	0.75 (0-2)	—	
**Emoji reactions**					
	Median	3 (1-6)	1 (0-2)	<.001	
	IQR	—	2 (1-4)	—	
**Engagements^c^**					
	Median	7 (3-12)	1.5 (0.5-2.5)	<.001	
	IQR	—	3.75 (1.75-6)	—	

^a^The Wilcoxon signed rank test was used to examine the difference between level 1 comments and matched comments and revealed that level 1 comments received more replies, emoji reactions, and engagements.

^b^Not available.

^c^Engagements = replies + emoji reactions.

In total, infodemiologists made 322 level 2 reply comments, precipitating 189 emoji reactions, of which 151 (79.9%) were positive (141 like, 10 love, and 0 hug emojis), 37 (19.6%) were negative (37 haha and 0 mad emojis), and 1 (0.5%) was neutral (1 wow and 0 sad emojis). The level 2 reply comments received a median of 0 replies with a median IQR of 0, 0 emoji reactions with a median IQR of 0, and 0.5 engagements with a median IQR of 0. The matched reply comments received a median of 0 replies with a median IQR of 0.75, 0.5 emoji reactions with a median IQR of 1, and 1 engagement with a median IQR of 2.5. Compared to the matched reply comments, the level 2 reply comments received fewer and narrower ranges of replies, reactions, and engagements, except for the median comparison for replies (Table 4).

**Table 4:** Metrics for infodemiologist intervention (level 2 reply comment) and matched reply comment.

			Level 2 reply comment, median (Q1-Q3)		Matched reply comment, median (Q1-Q3)		*P* value^a^		Poisson fixed-effects with robust SE estimates			Poisson random effects			Poisson random effects with the number of page followers/ 1,000,000 as an offset	
**Replies**										–0.98^b^			–0.98^b^			–0.99^b^
	Median	0 (0-0.5)		0 (0-0.25)		.94										
	IQR	0 (0-0.5)		0.75 (0-1.75)		<.001										
**Emoji reactions**										–1.19^b^			–1.21^b^			–1.20^b^
	Median	0 (0-0.5)		0.5 (0-1)		<.001										
	IQR	0 (0-0.3125)		1 (1-2.5)		<.001										
**Engagements** ^c^										–1.10^b^			–1.11^b^			–1.10^b^
	Median	0.5 (0-1)		1 (1-2)		<.001										
	IQR	0 (0-0.75)		2.5 (1-4.25)		<.001										

^a^Wilcoxon signed rank test.

^b^*P*<.001.

^c^Engagements = replies + emoji reactions.

The median number of individuals involved in conversation threads with the infodemiologist was 2 with a median IQR of 3. Qualitative evidence of psychological reactance or a backfire effect (assessed by observing if discussion with an infodemiologist appeared to immediately lead to a commenter leaving more extreme comments) was rare, appearing in 1% (2/145) of interventions.

## Discussion

The primary purpose of this research was to see if infodemiologist interventions would receive attention in web-based settings. We found that, by and large, they do; however, the evidence for such attention is limited due to Facebook’s data collection limitations, which require active engagement and do not provide data on the number of viewers that do not either comment or react using emojis. We also sought to develop a basis for assessing the extent of that attention by comparing engagement with our comments to engagement with comments made by others to the same post (matched comments). More specifically, on average, infodemiologists’ interventions (level 2 reply comments) received fewer replies and less overall sentiment (positive, neutral, or negative), as evidenced through native metrics such as “likes,” than matched reply comments. We found in this case that we had statistically significantly less engagement than the matched reply comments. Moreover, according to the IQR comparisons, we also observed that the infodemiologists’ comments received a narrower range of reactions, replies, and engagements than matched benchmark comments.

This could suggest that our impact was less than that of antivaccination comments, but another, more nuanced interpretation of our results, based on the context in which the interventions were made, is that our reply comments led to a quieting of the conversation rather than stimulating more antivaccination comments. On a highly charged political topic, such as the discourse surrounding COVID-19 vaccines, reducing engagement may be one effective way to reduce the spread of misinformation, even if the impact on participants’ and bystanders’ beliefs and behavior remains unclear. For example, numerous studies of accuracy nudges demonstrate such interventions reduce intentions to share misleading or false content [80-82].

We recognize that reduced engagement does not necessarily imply agreement. Moreover, there are several potential explanations for why infodemiologists comments received less engagement, including participant boredom, inattention, apathy, undetected or silent backfire effects, or algorithmic downregulation. However, Facebook data limitations preclude us from tracking the comments of either participants or bystanders after infodemiologist interventions to assess whether interventions changed their attitudes about vaccines or led to anyone’s decision to subsequently receive a vaccine.

While some engagement is useful or even necessary to algorithmically drive attention to infodemiologist interventions and therefore increase overall views, attempts to drive too much engagement—that is, through the strong emotional reactions of outrage or fear that may be required to drive such metrics—could be detrimental to the tenor of conversations infodemiologists are seeking to have. In addition, high degrees of engagement and the emotional valence such conversations are likely to bring may expose infodemiologists to other risks, such as harassment or doxing. The optimal amount of engagement that balances these 2 competing priorities is not clear.

Considering the optimal amount of engagement alludes to a larger issue about the metrics being used to assess such digital interventions overall. On social media, the metrics most convenient to use are designed for monitoring the impact of brand marketing [83,84]. Such metrics are not conducive to public health evaluations of the dynamics of misinformation [20]. Moreover, as has been previously noted, engagement itself does not necessarily align with efforts to prevent or mitigate the spread of misinformation about science and health. Efforts to stymie the production or spread of misinformation then face a strategic dilemma: maximize engagement through the native metrics made available by social media platforms (and incur the subsequent externalities) or engage in time- and labor-intensive practices of data collection to generate alternate metrics. The former approach implies that the solution to misinformation about science or medicine is simply to make science more engaging; however, as implied by Brandolini’s law that the time, effort, and cost of addressing falsehoods are orders of magnitude larger than the resources required to produce them [85], that approach ignores the diversity and ease with which misinformation about science spreads [86].

This study has several limitations. First and foremost are the limits on data accessibility that curtail efforts to fully understand the impact of infodemiology interventions on the digital information environment and other actors in this space. Indeed, various features of technology platforms’ algorithms and user interfaces made it challenging to maintain the same intervention strategy over time, follow ongoing interventions, and collect sufficient data at scale. This manifested as well in that Facebook approximates counts of comments and emojis once the number becomes large, leading to similar approximates in our reported data, especially for the number of page followers. Similarly, Facebook algorithms are constantly reordering comments in active threads. Therefore, the benchmarking data, which were collected after the original posting, may represent slightly different results than if the benchmarking were collected at the same time as the intervention itself. However, as the benchmarking is sufficiently broad, covering the comments both above and below the comment to which the infodemiologist replied, we believe it provides a representative “control” against which we can weigh the infodemiologists’ interventions.

Moreover, while third-party applications offer insight into “social media marketing metrics,” such services are structured to provide data to Facebook landing pages, not to assess engagement metrics of individuals doing multiple interventions across pages hosted by others. Specifically, because we did not own the pages on which we intervened, we could not access metrics on the views of those bystanders who neither commented nor provided emoji reactions through commercial software that permits such monitoring. Additionally, because engagement is defined as the combination of 2 nonmutually exclusive events (ie, emoji reactions and comments), it is possible that it is an overstatement of the number of people engaging in the discussion if someone both commented and expressed an emoji reaction.

Furthermore, it is impossible to separate the effect of the interventions completed by the infodemiologists from the potential impact of how they were viewed by other stakeholders in the discussion. For example, the perception that they may be trusted sources, researchers, or central nodes in a social network could influence how the interventions are received by participants. It is possible that infodemiologists were responding to, or the engagement numbers were inflated by, fake Facebook accounts. Infodemiologists sought to minimize this risk through the selection of which threads to intervene in and by examining a poster’s public profile. Additionally, this work does not address the myriad individual, community, and structural factors that lead to disparities in information sharing on social networks [87]. It also does not effectively address how the structure of the social network affects how health information diffuses [88]. Future work will seek to collect sufficient data to assess these potential confounders.

An additional challenge lies in how misinformation is defined. While our infodemiologists sought to respond to posts with factually incorrect material or overtly negative sentiment about the vaccines, those determinations were made based on context and therefore subjective. Moreover, the sheer volume of such material on social media meant infodemiologists could not respond to all such instances in their communities but only a subset.

Our sample was geographically skewed with relatively few New Jersey-focused interventions, we believe due to the overlap of New York and New Jersey media markets. Finally, our pilot prioritized infodemiologist safety and well-being both in the choice of the site of interventions (Facebook posts of news stories) and in the interventions themselves, instructing infodemiologists to avoid highly contentious forums and exit conversations if they felt threatened. It is likely that an explicit focus on engagement would lead to higher engagement statistics, though such approaches would need to be weighed against the externalities (eg, more contentious, emotionally laden content generating fear or outrage or the emotional safety or doxing of the infodemiologist).

Here we described an approach to addressing health-related misinformation derived from evidence on methods to intervene against misinformation about various topics in web-based and offline spaces that were associated with less engagement relative to comparable comments and replies in the same comment threads. More research building on some of the toolkits and frameworks presented here will be needed to further guide research on addressing misinformation in digital communities. We attempted to show that the RE-AIM framework is an effective schema to guide evaluations in this space, even if direct evidence of real-world efficacy is lacking. However, the gap between ideal evaluation metrics and the available data through social media platforms remains wide.

With this in mind, it raises the question of whether the inexorable drive for engagement—a metric prioritized by social media companies, not public health—is a solution to the problem of misinformation or further exacerbates it by not addressing the underlying mechanisms, incentives, and logic by which misinformation spreads. The infodemiology work described here and its impact on reducing the temperature of web-based conversations and avoiding backfire effects raise questions about how much engagement is optimal to improve science communication. More research is needed, of course, to correlate this approach with the effect on participants’ and bystanders’ subsequent beliefs and behaviors.

In previous reports, we examined our intervention protocol from several perspectives. In a study [30], we found a clear tension between using principles of motivational interviewing and the imperative to limit the amount of misinformation that remains unchecked by facts. Separately, we discussed that infodemiologists adopt several informal roles in web-based discussions, serving both as hosts and translators [22]. In this study, we quantitatively evaluated the impact of these interventions, drawing inspiration from implementation science frameworks as a guide, with the intention of understanding to what extent these interventions attract attention from bystanders. Viewed in combination, our qualitative analyses plus this quantitative assessment provide a novel mixed methods approach to evaluating interventions to address web-based antivaccine sentiment specifically and digital misinformation in general. Such approaches provide a more complete picture of the extent to which interventions based on a blend of motivational interviewing principles and evidence-based interventions focusing on bystanders can be useful in counteracting networked misinformation on web-based platforms. While labor-intensive, such interventions can be one part of a comprehensive strategy to address medical misinformation in digital spaces, along with other evidence-based strategies.

## Abbreviations

CDC: Centers for Disease Control and Prevention
IRB: institutional review board
MI: motivational interviewing
RE-AIM: reach, effectiveness, adoption, implementation, and maintenance
WHO: World Health Organization
